# Doctors

**Published:** 1876-07

**Authors:** 


### DOCTORS.

DR. NED.

When the last Oriental "Red Skin" retired with bow and arrow to his new hunting ground, "John Chinaman," without the least hesitation, put in his claim as the first and original discoverer of medicine.

Joseph, Moses, and Solomon had long worn professional honors without dispute, until "John" came armed with all the documents to render his position impregnable. "John" had in some way learned that the Egyptians had practised medicine seventeen hundred years before the Christian Era; he kept quiet until he was perfectly satisfied u pon the subject of antiquity, and then he introduced to the world "Hoam-ti," who ministered to the wants of suffering humanity, two thousand six hundred and eighty-seven years prior to the birth of Christ. In order to obviate all questions upon this matter, he produced the standard work of that early day entitled "Nuy'kine'" which even now is John's guide in the healing art. Some impudent fellow intimated that there was something wrong about the date of John's book, it was too long before the art of printing was discovered, and three hundred and thirty-eight years before the flood, in which all the inhabitants of the earth, except Noah and his family, were destroyed. Such a statement of facts would stagger some men, but no one ever saw a "Celestial" intimidated. As soon as the *big rain* was referred to, "John" produced a minute biography of Noah, containing a full account of the storm, with all the incidents of his journey, written by himself, including a statement of his business transactions with dealers in teas at Hong Kong, for three hundred and fifty-one years and four months after he abandoned the occupation of a sailor, during which time he rented the ark for a fishing craft.

"John" places the utmost reliance upon the pulses; he examines them all; the supreme or "celestial" pulse, placed at the articulation of the fore-arm with the wrist, indicates to him the condition of all the organs from the head to the epigastrium; the pulse in the right arm tells the state of the heart, while in the left, it shows the condition of the lungs; and the terrestial pulse, situated at the articulation of the wrist with the hand, indicates the moisture or dryness of the system.

He examines all these, nine in number, acting as if he was playing the piano, and when the tune is ended, gives his opinion without the least hesitation, makes his prescription, takes his money, *always*, and retires! He knows how important it is to seem wise and mysterious; accordingly he assumes great wisdom and profound learning, as much so as any American; he compresses his lips, opens his eyes as wide as he can stretch them, runs the fingers of his left hand rapidly over his scalp, as if in quest of something the comb left, and proceeds to explain the wonderous analogy that exists between fire and the heart, the planet *Mars*, the poker, the mother-in-law's tongue, and everything else that is supposed to be hot; he is perfectly familiar with forty-two varieties of varioloid and twice that number of distinct forms of the measels! He has a name for every ache and pain, and gives the cause of every disturbance that occurs between the cradle and the grave, including "family jars." No person can say or do more than he, and of course his wisdom finds a ready market amongst our afflicted people, who purchase his compounds at fearful cost, not omitting a supply of his "ginseng root," to reanimate the vital forces and drive away the infirmities of age.

At one time, in China, they had laws prohibiting any, except those who had passed through medical schools, to follow the profession, but now such foolish enactments no longer exist, and anyone who wishes can administer to the sick, for, Americans have gone there and carried with them the liberal ideas of an advanced civilization and freedom.

In the dark ages of the Republic our laws were so framed that only such as were by education entitled to trust and confidence could enter the sick room as medical advisers. We had not then fully comprehended the meaning of personal freedom; it is true we professed to be free; and all the time we were so ignorant concerning the benefits that freedom carried with it, that we submitted to unjust and arbitrary statutes through long tedious years; but *now*, all the obstacles are removed and everybody, high and low, black and white, learned or unlearned, can vomit, or be vomited, without distinction or restraint; now, to physic or be physiced, to be bled, blistered, butchered, killed or cured, is a National right that belongs to every free American, and one that he enjoys; it is right, and should be maintained at all hazards.

If a man desires to become a banker, he must obtain a charter for his institution, which is just, because he is dealing in money that may belong to other persons, and, besides, if he should cheat his depositor, others might suffer, and *everybody* could find it out, but such is not the case with Doctors. A man's health is his own; he has a family of his own, and if he sees fit to ruin his health, *his own* can have a first class chance to practice the Christian Virtues and take care of him. If he destroys his life, why, he may have an unoccupied cemetery lot all paid for, and it is no one's business if he takes personal possession of his own premises; and if he is disposed to be charitable and contributes of his means to the support of some ignoramus, who is generous with the printer, no person should interfere if he gets killed, because it would never get out; all honest, sincere friends could mourn, some of his near relatives would put on black for a season, and good people would exclaim, "the will of the Lord be done." If a man wants to practice the Law, he must have the proper credential before he can participate in a contest involving the life of a dog, that is worthless, because he will not bite and should be killed if he does. The dealer in liquors must have his license before he can administer medicated drinks to his patients, and that is right, for he does not make use of doses large and powerful enough to lay his customers *out*, so that they are harmless; he gives them just enough to make them wild, crazy and dangerous, unless they fall into the hands of some liberal unlicensed Doctor, in which case they soon become as docile as lambs, and as manageable as babies. If they have any kind of luck, wind up by giving the neighborhood a first-class entertainment, conducted by the undertaker at exhorbitant prices. If a man has a fancy dog, or a good horse, or a mule, of reasonable back-action proclivities, that is sick, he will make every effort to secure the best skill he can obtain, in order to save the brute; every interest impels him thus to act, and besides, there is a law prohibiting cruelty to animals which will ere long give way to the progress and culture of this enlightened age, without doubt. If man was considered an animal, and subject to the same arbitrary exacting protection, it would destroy a very large extended and profitable business throughout the land, and throw thousands of men out of employment, who are now engaged in the humane enterprises of making pills, plasters, potions, physics, infusions, tinctures, syrups, decoctions, emetics, resolvents, diuretics and compounds without number, to be taken by mankind, so that nurses, doctors and gravediggers can be kept at work, and in this way every department of business is encouraged and every variety of talent employed. Agents kindly and politely bring the charming productions to our doors, and many forego the comforts of home and all the endearing associations of early life, becoming missionaries among their fellows, they travel from place to place and at enormous cost, publish to the world their wonderful cures and their Samaritan labors. In this way, they add to the world's wealth, increase the demand for stone-cutters, and improve, as well as add, uncultivated acres to cemeteries; all these I am sure are indications of energy and thrift, as privileges that thousands and tens of thousands have sacrificed their lives to maintain and defend.

If it is right and judicious to take some physic from some persons, then all should enjoy the luxury of taking every kind, from everybody. Let us hope that the day will come when the State will act wisely and well by properly rewarding the vendors of physic, the patentees of pills, and the swarms of traveling medical colporters, by giving them a chance to develop their muscles and rest their heads while engaged in the non-honorable employment of working for the State, which honors them with a uniform, to which, if the stars were added, they would look like so many flags at half mast.

